# Bedquiline Resistance Mutations: Correlations with Drug Exposures and Impact on the Proteome in M. tuberculosis

**DOI:** 10.1128/aac.01532-22

**Published:** 2023-05-31

**Authors:** Jian Xu, Dongshuo Li, Jinghua Shi, Bin Wang, Fei Ge, Zhenyong Guo, Xiaopan Mu, Eric Nuermberger, Yu Lu

**Affiliations:** a Beijing Key Laboratory of Drug Resistance Tuberculosis Research, Beijing Tuberculosis and Thoracic Tumor Research Institute, and Beijing Chest Hospital, Capital Medical University, Beijing, China; b Center for Tuberculosis Research, Department of Medicine, Johns Hopkins University School of Medicine, Baltimore, Maryland, USA; c Department of International Health, Johns Hopkins Bloomberg School of Public Health, Baltimore, Maryland, USA

**Keywords:** *Mycobacterium tuberculosis*, *Rv0678*, *atpE*, bedaquiline, exposure, proteome, resistance

## Abstract

Bedaquiline (BDQ) is an effective drug for the treatment of drug-resistant tuberculosis. Mutations in *atpE*, which encodes the target of BDQ, are associated with large increases in MICs. Mutations in *Rv0678* that derepress the transcription of the MmpL5-MmpS5 efflux transporter are associated with smaller increases in MICs. However, *Rv0678* mutations are the most common mutations that are associated with BDQ resistance in clinical isolates, and they also confer cross-resistance to clofazimine (CFZ). To investigate the mechanism of BDQ resistance and the correlation between *Rv0678* mutations and target-based *atpE* mutations, M. tuberculosis strains were exposed to different concentrations of BDQ or CFZ to select *Rv0678* mutations and *atpE* mutations. Gene overexpression strains were constructed to illustrate the roles of MmpL5 and MmpS5. A quantitative proteome analysis was performed to compare the BDQ-resistant mutants to the isogenic strain H37Rv. Here, we report that the *Rv0678* mutations were more readily selected than were the *atpE* mutations at low concentrations of BDQ or CFZ. The *atpE* mutations were selected by high concentrations of BDQ exposure. The overexpression of both *mmpL5* and *mmpS5* reduced the susceptibility of Mycobacterium tuberculosis to BDQ and CFZ. Secreted immunogenic proteins and proteins involved in the biosynthesis and transport of phthiocerol dimycocerosates were associated with *Rv0678* mutations conferring BDQ resistance in the proteome analysis. In conclusion, exposure to different bedaquiline concentrations resulted in the selection of different mutations. The coexpression of MmpL5 and MmpS5 contributed to drug resistance and upregulated pathogenic proteins in M. tuberculosis, suggesting MmpL5-MmpS5 as a new potential target for antituberculosis drug development. These results warrant further surveillance for the evolution of BDQ resistance during clinical usage.

## INTRODUCTION

Tuberculosis (TB), which is caused by Mycobacterium tuberculosis, was the second leading infectious killer after coronavirus disease 2019 (COVID-19) in 2020 and 2021. The World Health Organization (WHO) estimated that 10.6 million people fell ill with TB worldwide in 2021 and that 1.6 million people died from TB (1). Drug-resistant TB is a major threat, and about half a million people fall ill with drug-resistant TB annually (1). Bedaquiline (BDQ) was approved for the treatment of multidrug-resistant tuberculosis (MDR-TB), as the first new TB drug in over 4 decades (2), and it is now classified as a group A drug (to be included in all drug-resistant TB regimens) by the WHO (3). Clofazimine (CFZ), which is a riminophenazine drug, has been commonly used in the treatment of leprosy. Recently, CFZ was repurposed as an important component of a new, short-course MDR-TB regimen (4, 5). The WHO has recommended CFZ as a preferred agent for the treatment of drug-resistant TB (1) and has classified CFZ as a group B drug (3).

Alarmingly, M. tuberculosis strains with BDQ resistance were identified soon after the introduction of BDQ (6–8). BDQ inhibits mycobacterial ATP synthesis by targeting subunit C of the ATP synthase encoded by the *atpE* gene (9). BDQ resistance is conferred by target mutations in the *atpE* gene (9), as well as by nontarget mutations in *Rv0678* (10) and *pepQ* (11). Mutations in *Rv0678*, which encodes a transcriptional repressor of the MmpS5-MmpL5 efflux system, were shown to cause cross-resistance between BDQ and CFZ due to an upregulation of MmpL5/MmpS5 efflux transporter expression (6, 10). *Rv0678* mutations are the major mechanism of BDQ resistance among clinical isolates (6, 12–16), including primary BDQ and CFZ resistance among clinical isolates from TB patients without known prior exposure to BDQ or CFZ (12, 16, 17). Mutations in the putative proline aminopeptidase gene *pepQ* have rarely been associated with BDQ resistance in clinical isolates (14, 15).

As *Rv0678* mutations confer lower MIC values for BDQ than do *atpE* mutations in M. tuberculosis, and as *Rv0678* mutations have been identified in drug-resistant isolates that were obtained prior to the introduction to BDQ or the use of CFZ for TB, an important question that needs to be answered is whether *Rv0678* mutations appear as precursors to the high-level resistance that is caused by *atpE* mutations. The concept of the stepwise acquisition of drug-resistance mutations conferring additive, selective advantages through greater drug resistance and/or greater fitness is supported by examples of sequential *inhA* promoter and *katG* mutations conferring additive resistance and the acquisition of compensatory *rpoB* or *rpoC* mutations that enhance the fitness (and sometimes the resistance) of *rpoB* mutants. One example is a study of the evolution of *rpoB* mutations in South Africa, in which secondary *rpoB* mutations were posited to contribute to the increased transmission and dispersal of the KZN/LAM4 strain via enhanced resistance to rifampin and fitness, compared to the isolated, low-level rifampin resistance mutation in L452P (18).

The goals of our study were to investigate whether low-level *Rv0678*-mediated BDQ resistance is a stepping stone to high-level *atpE* mediated resistance and to explore the changes in relative protein abundance that were correlated with BDQ resistance mutations. First, we compared the spontaneous frequency of resistance to BDQ in a clinical isolate from a major phylogenetic lineage (lineage 2) to that of the H37Rv strain (lineage 4). Then, we identified the relationship between BDQ or CFZ exposure and the acquisition of *Rv0678* and *atpE* mutations. Next, we investigated the roles of MmpL5 and MmpS5, as regulated by *Rv0678* in M. tuberculosis (MTB). Finally, a proteome analysis was conducted to investigate the protein abundance associated with BDQ resistance mutations.

## RESULTS

### A lower frequency of spontaneous bedaquiline resistance mutations in *Rv0678* is observed in a clinical isolate from the Beijing family.

To compare the spontaneous frequency of resistance to BDQ in a clinical isolate from another major phylogenetic lineage (i.e., lineage 2) to that of the H37Rv strain (lineage 4), we plated log-phase cultures of M. tuberculosis H37Rv and the BDQ-susceptible XDR-TB clinical isolate 16833 with the Beijing family genotype on agar containing 0.125, 0.25, 0.5, and 1 μg/mL of BDQ. The spontaneous frequency of resistance to BDQ at 0.125 μg/mL in isolate 16833 was about 10-fold lower (8.4 × 10^−7^) than that observed in H37Rv (8.0 × 10^−6^). Similarly, the spontaneous frequency of resistance to BDQ at 0.25 and 0.5 μg/mL in isolate 16833 (3.6 × 10^−7^ and 8.6 × 10^−8^) was approximately 10-fold lower than that observed in H37Rv (2.6 × 10^−6^ and 8.1 × 10^−7^), respectively. Two colonies of each strain were picked from plates containing 0.125, 0.25, or 0.5 μg/mL BDQ for the sequencing of *Rv0678* and *atpE* (Table 1). Only 1 colony growing on a 1 μg/mL BDQ-containing plate was found and sequenced in strain 16833 and H37Rv.

**TABLE 1:** Spontaneous mutations observed at different concentrations of BDQ in strain 16833 and H37Rv^*a*^

Isolates	*Rv0678*	*atpE*	MIC (μg/mL)	
			BDQ	CFZ
16833 0.125B-1	g137 insertion	WT	0.56	2.15
16833 0.125B-2	WT	WT	-	-
16833 0.25B-1	c247g	WT	0.49	0.49
16833 0.25B-2	c466 insertion	WT	0.6	1.78
16833 0.5B-1	c244t	WT	0.5	1.56
16833 0.5B-2	g193 insertion	WT	0.95	0.97
16833 1B-1	g52t	WT	0.96	0.47
16833	WT	WT	0.05	0.14
Rv 0.125B-1	g193 insertion	WT	0.48	1.63
Rv 0.125B-2	t298g	WT	-	-
Rv 0.25B-1	c305t	WT	-	-
Rv 0.25B-2	t298g	WT	-	-
Rv 0.5B-1	g193 deletion	WT	-	-
Rv 0.5B-2	t298g	WT	-	-
Rv 1B-1	t298g	WT	0.49	0.98
H37Rv	WT	WT	0.063	0.24

a -, not determined. *Rv0678* mutations with a g137 insertion, c466 insertion, g193 insertion, c305t and g193 deletion have also been identified in clinical isolates.

Isogenic *Rv0678* mutants in both strain backgrounds were cross-resistant to BDQ and CFZ. Mutations were scattered throughout the *Rv0678* gene, although one common mutation with a g193 insertion in *Rv0678* was found in both strains. Although *Rv0678* t298g mutants were selected in H37Rv from plates containing 0.125, 0.25, or 0.5 μg/mL BDQ, there is no report in clinical isolates of M. tuberculosis so far. No mutations were found in *atpE* among the BDQ-resistant strains.

### The acquisition of *Rv0678* mutations is more frequent than the acquisition of *atpE* mutations at low selective concentrations of BDQ or CFZ.

To clarify the relationship between BDQ or CFZ exposure and the acquisition of *Rv0678* and *atpE* mutations, approximately 10^7^ CFU/mL of M. tuberculosis H37Rv, an *Rv0678* mutant in the H37Rv strain background with a g193 insertion, and the clinical strain 16833 were exposed to 0.03 μg/mL (1/2× MIC) BDQ or 0.12 μg/mL (1/2× MIC) CFZ for 3 weeks in 7H9 medium. Cultures were plated on drug-free agar and agar containing BDQ at 0.125 μg/mL and 1 μg/mL. Two colonies in each group at each BDQ plate concentration were picked for the sequencing of *Rv0678* and *atpE*. Compared to H37Rv, strain 16833 had about a 4-fold lower frequency of resistance to 0.125 μg/mL BDQ after exposure to 0.03 μg/mL BDQ or 0.12 μg/mL CFZ (Table 2). Among the three strains exposed to BDQ or CFZ, only one *atpE* mutant with a c188t mutation was isolated from the isogenic *Rv0678* mutant background. Although one colony with the *Rv0678* mutation background grew on the BDQ-containing plates at 1 μg/mL after exposure to CFZ, no *atpE* mutation was found. The wild type *Rv0678* strains H37Rv and 16833 had no *atpE* mutation after incubation with 0.03 μg/mL BDQ or 0.12 μg/mL CFZ.

**TABLE 2:** *Rv0678* and *atpE* mutations accumulated over 3 weeks of incubation with 0.03 μg/mL BDQ or 0.12 μg/mL CFZ as well as the frequencies at which they appeared

Property	M. tuberculosis strain^*a*^ (baseline genotype [*Rv0678*, *atpE*]) and drug exposure					
	H37Rv (WT, WT)		*Rv0678* mutant (g193 insertion, WT)		Strain 16833 (WT, WT)	
	BDQ	CFZ	BDQ	CFZ	BDQ	CFZ
	(0.03 μg/mL)	(0.12 μg/mL)	(0.03 μg/mL)	(0.12 μg/mL)	(0.03 μg/mL)	(0.12 μg/mL)
Log_10_CFU/mL count on drug-free agar	7.48	7.18	7.73	7.56	8	7.9
Log_10_CFU/mL count on agar with BDQ 0.125 μg/mL (*Rv0678*, *atpE*)^*b*^	3.3 (t284c, WT)^1/2^; (c141 insertion, WT)^1/2^	3.34 (c141 insertion, WT)^1/2^; (g193 insertion, WT)^1/2^	7.73 (g193 insertion, WT)^2/2^	7.56 (g193 insertion, WT)^2/2^	3.18 (c141 insertion, WT)^1/2^; (t416c, WT)^1/2^	3.48 (c244t, WT)^1/2^; (c244t, WT)^1/2^

Resistance frequency at 0.125 μg/mL BDQ	6.7 × 10^−5^	1.5 × 10^−4^	100%	100%	1.5 × 10^−5^	3.8 × 10^−5^
CFU/mL count on agar with BDQ 1 μg/mL (*Rv0678*, *atpE*)	0	0	1 (g193 insertion, c188t)	1 (g193 insertion, WT)	0	0
	

a The MICs of BDQ against the initial strain H37Rv, *Rv0678* mutant, and 16833 were 0.06, 0.48, and 0.06 μg/mL, respectively.

b Superscript numbers indicate the proportion of colonies with the indicated genotype among the colonies tested.

As only a single resistant colony with an *atpE* mutation was isolated when there were more CFU plated for the *Rv0678* mutant, compared to H37Rv, the experiment was repeated with a higher bacterial burden of M. tuberculosis H37Rv and *Rv0678* mutant being exposed to 0.03 μg/mL (1/2× MIC) or 0.3 μg/mL (5× MIC) BDQ for 4 weeks in 7H9 medium. Additionally, the clinical strain 16833 and two clinical strains with *Rv0678* mutations (12657, 13476) were exposed to 0.03 μg/mL BDQ for 4 weeks. There was no bacterial growth on 1 μg/mL BDQ-containing plates in all five of the tested strains after exposure to 0.03 μg/mL BDQ (Table 3). However, 6 out of 272 colonies growing on the 1 μg/mL BDQ-containing plates after H37Rv exposure to 0.3 μg/mL BDQ harbored a g187c mutation in *atpE* without a mutation in *Rv0678*. Out of two colonies growing on 1 μg/mL BDQ-containing plates after the *Rv0678* mutant exposure to 0.3 μg/mL BDQ, one colony had a g187c mutation in *atpE*. Consistent with the results of the previous experiment, the colonies growing on the 0.125 μg/mL BDQ-containing plates that were inoculated with clinical isolate 16833 after exposure to 0.03 μg/mL BDQ had *Rv0678* mutations with no mutation in *atpE*. There were no new mutations in *Rv0678* in clinical isolates 12657 and 13476 after exposure to 0.03 μg/mL BDQ for 4 weeks.

**TABLE 3:** *Rv0678* and *atpE* mutations accumulated over 4 weeks of incubation with 0.03 μg/mL BDQ or 0.3 μg/mL BDQ

Property	M. tuberculosis strain^*a*^ (baseline genotype [*Rv0678*, *atpE*]) and drug exposure								
	H37Rv (WT, WT)			*Rv0678* mutant (g193 insertion, WT)			Strain 16833 (WT, WT)	Strain 12657 (c158t, WT)	Strain 13476 (t350g, WT)
	Untreated	0.03 μg/mL BDQ	0.3 μg/mL BDQ	Untreated	0.03 μg/mL BDQ	0.3 μg/mL BDQ	0.03 μg/mL BDQ	0.03 μg/mL BDQ	0.03 μg/mL BDQ
Log_10_CFU/mL count on drug-free agar	7.88	8.16	7.63	8.28	8.49	8.47	9.1	8.7	8
Log_10_CFU/mL count on agar with 0.125 μg/mL BDQ (*Rv0678*, *atpE*)^*b*^	ND	ND	ND	ND	ND	ND	4 (g193 insertion, WT)^1/2^; (c251t, WT)^1/2^	7.8 (c158t, WT)^2/2^	7.3 (t350g, WT)^2/2^
CFU/mL count on agar with 1 μg/mL BDQ (*Rv0678*, *atpE*)^*a*^	0	0	272 (WT, g187c)^6/6^	0	0	2 (g193insertion, g187c)^1/2^;(g193insertion, WT)^1/2^	0	0	0

a The MICs of BDQ against the initial strain H37Rv, *Rv0678* mutant, 16833, 12657, and 13476 were 0.06, 0.48, 0.06, 0.39, and 0.44 μg/mL, respectively.

b Superscript numbers indicate the proportion of colonies with the indicated genotype among the colonies tested. ND, not done.

### Exposure to a high concentration of BDQ is linked with the acquisition of *atpE* mutations.

To determine whether the low-level resistance caused by *Rv0678* mutations could be overcome via treatment with high concentrations of BDQ or CFZ, approximately 10^7^ CFU/mL of MTB H37Rv, an *Rv0678* mutant with a g193 insertion, and strain 16833 were exposed to 30 μg/mL BDQ or 120 μg/mL CFZ for 4 weeks in 7H9 medium. As expected, no bacteria survived after exposure to a concentration 500 times higher than the MIC of CFZ in these 3 strains (Table 4). To our surprise, the *Rv0678* mutant grew well, despite exposure to a concentration 500 times higher than the wild-type MIC of BDQ, and it had acquired an a83g mutation in *atpE*. Although we did not plate the bacteria on drug-free agar, the liquid cultures of H37Rv and strain 16833 were clear, and the liquid culture of *Rv0678* mutant was turbid. A few colonies with a g187c *atpE* mutation were isolated from the wild-type H37Rv strain that was plated on 0.125 μg/mL BDQ-containing plates (2 colonies) and on 1 μg/mL BDQ-containing plates (19 colonies). No growth was observed after strain 16833 was exposed to the high concentration of BDQ.

**TABLE 4:** *atpE* mutations accumulated over 4 weeks of incubation with 30 μg/mL BDQ or 120 μg/mL CFZ

Property	M. tuberculosis strain^*a*^ (baseline genotype [*Rv0678*, *atpE*]) and drug exposure					
	H37Rv (WT, WT)		*Rv0678* mutant (g193insertion, WT)		Strain 16833 (WT, WT)	
	BDQ 30 μg/mL	CFZ 120 μg/mL	BDQ 30 μg/mL	CFZ 120 μg/mL	BDQ 30 μg/mL	CFZ 120 μg/mL
CFU/mL count on agar with BDQ 0.125 μg/mL (*Rv0678*, *atpE*)^*b*^	2 (WT, g187c)^2/2^	0	Many (>1000) (g193 insertion, a83g)^2/2^	0	0	0
CFU/mL count on agar with BDQ 1 μg/mL (*Rv0678*, *atpE*)^*b*^	19 (WT, g187c)^2/2^	0	Many (>1000) (g193 insertion, a83g)^2/2^	0	0	0

a The MICs of BDQ against the initial strain H37Rv, *Rv0678* mutant, and 16833 were 0.06, 0.48, and 0.06 μg/mL, respectively.

b Superscript numbers indicate the proportion of colonies with the indicated genotype among the colonies tested.

### A genome analysis confirmed no other mutations, except *Rv0678*, *atpE* or both genes.

To identify other mechanisms of resistance in BDQ-resistant isolates, we subjected the four isogenic BDQ-resistant strains that were selected in the preceding experiments (Tables 2 and 4; strains D1, D12, H3, and L11 in Table 5) as well as strain H37Rv to whole-genome sequencing. The dual mutants D1 and D12 had a g193 insertion in *Rv0678* and *atpE* mutations at c188t and a83g, respectively. The mutant H3 had an g187c *atpE* mutation alone, and the mutant L11 had an *Rv0678* mutation at the g193 insertion alone. A comparative genome sequence analysis confirmed the *Rv0678* and *atpE* mutations that were previously identified by PCR-based sequencing in the four BDQ-resistant strains (D1, D12, H3, and L11) as well as the absence of mutations in H37Rv (Table 5). No other common mutations were found via single nucleotide polymorphism (SNP) and insertion-deletion (Indel) analyses, including in the *pepQ* and *Rv1979c* genes that were previously associated with resistance. The RT-PCR results showed the upregulated expression of *mmpS5-mmpL5* in the *Rv0678* mutants (D1, D12, and L11) but not in strain H3 with an *atpE* mutation alone. The MIC results demonstrated that *atpE* mutations are associated with high-level resistance (BDQ MIC of >1.5 μg/mL), compared to *Rv0678* mutations alone (BDQ MICs of <1 μg/mL). As expected, strain H3 with an isolated *atpE* mutation did not have an elevated CFZ MIC.

**TABLE 5:** Drug susceptibility profiles and gene expression of BDQ-resistant M. tuberculosis

Strains	*Rv0678*	*atpE*	*pepQ*	Fold change in expression			BDQ (μg/mL)	CFZ (μg/mL)
				*mmpL5*	*mmpS5*	*Rv0678*		
D1	g193 insertion	c188t	WT	5.42	2.64	1.29	1.59	1.33
D12	g193 insertion	a83g	WT	8.77	5.41	3.74	2.07	1.99
H3	WT	g187c	WT	1.58	1.29	1.87	1.91	0.24
L11	g193 insertion	WT	WT	10.24	5.1	5.73	0.48	1.63
H37Rv	WT	WT	WT	-^*a*^	-	-	0.063	0.24

a -, not determined.

### The overexpression of both *mmpL5* and *mmpS5* is required to increase the BDQ and CFZ MICs.

To identify the roles of MmpS5 and MmpL5 in the resistance to BDQ and CFZ in MTB, we constructed three plasmids encoding either MmpS5, MmpL5, or MmpS5-MmpL5 with the plasmid pMV361. MTB H37Rv cells were transformed with these plasmids and the control plasmid pMV361. The RT-PCR results confirmed the overexpression of *mmpS5*, *mmpL5*, and *mmpS5-mmpL5*, respectively (Table 6). The MICs of BDQ and CFZ against each strain were determined (Table 6). The strain overexpressing both *mmpS5* and *mmpL5* exhibited substantially higher MICs for BDQ (13-fold) and CFZ (6-fold). However, overexpressing *mmpS5* or *mmpL5* separately did not affect the MICs of BDQ and CFZ. These results show that *mmpS5* and *mmpL5* must be overexpressed together to confer resistance to BDQ and CFZ.

**TABLE 6:** Expression of the *mmpL5-mmpS5* efflux pump in overexpression strains and its MICs

Strain	Fold change (RT-PCR)			MIC (μg/mL)	
	*mmpL5*	*mmpS5*	*Rv0678*	BDQ	CFZ
pMV361-S5-3	0.54	1.96	1.72	0.04	0.19
pMV361-L5-3	5.51	0.34	1.69	0.045	0.29
pMV361-S5-L5-3	3.95	2.85	2.79	0.48	1.28
pMV361-H37Rv	-^*a*^	-	-	0.055	0.21

a -, not determined.

### M. tuberculosis proteins associated with BDQ resistance.

To investigate the changes in protein abundance that are associated with BDQ resistance mutations, we performed a quantitative whole proteome analysis to compare the mutants D1, H3, L11 and D12 to the isogenic strain H37Rv. Using a tandem mass tag (TMT) labeling approach, coupled with high-resolution mass spectrometry, we identified 2,923 proteins that could be quantified to determine the relative protein expression. The top upregulated proteins in the *Rv0678* mutants and strains with combined mutations of *Rv0678* and *atpE* genes, relative to H37Rv, are shown in Tables 7 and 8, respectively. The downregulated proteins in the corresponding groups are shown in Table S2 and in Table S3, respectively. The top upregulated and downregulated proteins in the *atpE* mutant (H3), compared to H37Rv, are shown in Table 9 and in Table S4, respectively. As expected, the MmpS5 and MmpL5 proteins were more than twofold upregulated in the strains (L11, D1, and D12) with *Rv0678* mutations, compared to the parent strain H37Rv (Table 7). As expected, the expression of MmpS5 and MmpL5 in the *atpE* mutant H3 was not significantly different, compared to H37Rv. Other major upregulated proteins in the *Rv0678* mutants were secreted immunogenic proteins (MPT64, MPT70, Apa, espA, CFP-2, and Ag85B), which are known mycobacterial adhesins and proteins that contribute to the intracellular survival of M. tuberculosis (19) as well as proteins that are involved in the biosynthesis and transport of phthiocerol dimycocerosates (PDIM): PpsB, PpsC, PpsD, and DrrB (20). In all four BDQ-resistant strains with *Rv0678* (L11), *atpE* (H3), or both genes mutated (D1 and D12), other major upregulated proteins included chp^2^, SodB, the nucleoid-associated global regulatory proteins HupB and mIHF, and the ribosomal proteins RpmF, RpmJ, and RpsT (Table 8). chp^2^ is involved in the final steps of polyacyltrehalose (PAT) biosynthesis, and its activity is probably potentiated by MmpL10 (21). In the BDQ-resistant strains with the *atpE* mutation only (H3), but not the *Rv0678* mutant L11 (Table 9), the major upregulated proteins (GpsA, GlgM, and Ald) were related to the central carbon metabolism.

**TABLE 7:** Top 15 upregulated proteins with the highest average fold changes across the 3 *Rv0678* mutants L11, D1, and D12, but not H3, compared to H37Rv

Rv no.	Gene	Description	Fold change L11/Rv	Fold change H3/Rv	Fold change D1/Rv	Fold change D12/Rv
Rv1906c	*Rv1906c*	Conserved protein	6.6	1.3	2.8	3.6
Rv0061c	*Rv0061c*	Uncharacterized protein	3.8	1.1	2.5	2.9
Rv0677c	*mmpS5*	Siderophore export accessory protein MmpS5	3.6	1.1	2.9	2.9
Rv2937	*drrB*	Doxorubicin resistance ABC transporter permease protein DrrB	3.6	1.4	2	2.8
Rv1980c	*mpt64*	Immunogenic protein MPT64	3.4	1.3	2.8	3
Rv2875	*mpt70*	Immunogenic protein MPT70	3	1.2	3.1	1.9
Rv2576c	*Rv2576c*	Uncharacterized protein Rv2576c	2.8	1.2	2.2	2.1
Rv1860	*apa*	Alanine and proline-rich secreted protein Apa	2.4	1.1	1.8	1.9
Rv3616c	*espA*	ESX-1 secretion-associated protein EspA	2.3	1.2	1.8	2.3
Rv2376c	*cfp2*	Low mol wt antigen MTB12	2.2	1.1	1.6	1.9
Rv2932	*ppsB*	Phenolphthiocerol/phthiocerol polyketide synthase subunit B	2.2	1	1.8	1.8
Rv2934	*ppsD*	Phenolphthiocerol/phthiocerol polyketide synthase subunit D	2.1	1	1.6	1.8
Rv1886c	*fbpB*	Diacylglycerol acyltransferase/ mycolyltransferase Ag85B	2.1	1.3	2.2	2.5
Rv0676c	*mmpL5*	Siderophore exporter MmpL5	2.1	1	2.3	2.2
Rv2933	*ppsC*	Phenolphthiocerol/phthiocerol polyketide synthase subunit C	2.0	1.0	1.5	1.6

**TABLE 8:** Top 15 upregulated proteins with the highest average fold changes across all 4 BDQ-resistant strains H3, L11, D1, and D12, compared to H37Rv

Rv no.	Gene	Description	Fold change H3/Rv	Fold change L11/Rv	Fold change D1/Rv	Fold change D12/Rv
Rv1184c	*chp^2^*	Diacyltrehalose acyltransferase Chp2	3.0	1.8	1.6	1.9
Rv3878	*espJ*	ESX-1 secretion-associated protein EspJ	2.7	4.2	2.7	2.8
Rv0083	*Rv0083*	Uncharacterized protein Rv0083	2.6	2.4	2.3	2.4
Rv3846	*sodB*	Superoxide dismutase [Fe]	2.6	3.0	2.9	1.9
Rv2986c	*hupB*	DNA-binding protein HU homolog	2.5	2.6	1.9	2.0
Rv3678A	*Rv3678A*	Uncharacterized protein	2.5	2.8	2.2	1.9
Rv1580c	*Rv1580c*	Probable PhiRv1 phage protein	2.4	3.0	2.2	2.0
Rv3835	*Rv3835*	Uncharacterized membrane protein Rv3835	2.2	1.8	1.6	1.7
Rv1682	*Rv1682*	Probable coiled-coil structural protein	2.1	3.3	3.1	2.5
Rv1135c	*PPE16*	Uncharacterized PPE family protein PPE16	2.1	2.5	2.0	2.4
Rv1388	*mihF*	Putative integration host factor mIHF	2.0	2.2	1.8	1.9
Rv0979A	*rpmF*	50S ribosomal protein L32	2.0	3.8	2.4	2.7
Rv3461c	*rpmJ*	50S ribosomal protein L36	2.0	2.7	1.9	1.9
Rv3190A	*Rv3190A*	Conserved protein	2.0	3.0	2.0	1.9
Rv2412	*rpsT*	30S ribosomal protein S20	1.9	3.8	2.5	2.8

**TABLE 9:** Top 10 upregulated proteins with the highest average fold changes in the *atpE* mutant only (H3), but not in the *Rv0678* mutant L11, compared to H37Rv

Rv no.	Gene	Description	Fold change H3/Rv	Fold change L11/Rv	Fold change D1/Rv	Fold change D12/Rv
Rv1566c	*Rv1566c*	Possible Inv protein	2.9	1.1	1.6	1.3
Rv2982c	*gpsA*	Glycerol-3-phosphate dehydrogenase (NAD[P]+)	2.3	1.2	1.2	1.2
Rv0186A	*mymT*	Metallothionein	2.2	1.3	1.1	1.3
Rv1212c	*glgM*	Alpha-maltose-1-phosphate synthase	2.1	1.2	2.7	1.4
Rv0968	*Rv0968*	Uncharacterized protein Rv0968	1.9	1.4	2.3	1.8
Rv2190c	*Rv2190c*	Probable endopeptidase Rv2190c	1.8	1.1	2.9	1.2
Rv2780	*ald*	Alanine dehydrogenase	1.8	1.4	1.9	1.4
Rv1196	*PPE18*	PPE family protein PPE18	1.8	1.2	2.2	1.6
Rv3865	*espF*	ESX-1 secretion-associated protein EspF	1.8	1.4	2.2	1.5
Rv2577	*Rv2577*	Uncharacterized protein Rv2577	1.7	1.1	1.2	1.2

## DISCUSSION

In this study, the spontaneous frequency of resistance to BDQ in clinical isolate 16833 was about 4- to 10-fold lower than that observed in H37Rv on agar containing BDQ from 0.125 to 0.5 μg/mL. Although mutant frequencies were compared in only two strains, we obtained similar proportions (10-fold differences) in the spontaneous frequencies of resistance to BDQ at three different concentrations (0.125, 0.25, and 0.5 μg/mL). Furthermore, the two strains had about a 4-fold lower frequency of resistance to 0.125 μg/mL BDQ after 3 weeks of exposure to 0.03 μg/mL BDQ. A limitation of this study is that only one strain from each lineage was evaluated. More strains should be investigated to compare the mutation frequencies in the different lineages in a future study. Although the mutations were scattered across the *Rv0678* gene, the g193 insertion appeared to be a relative hot spot for mutations in *Rv0678.* It was selected from our clinical isolate 16833 and from H37Rv on BDQ-containing agar after exposure to either BDQ or CFZ-containing agar (22). As H37Rv is a lineage 4 strain and clinical isolate 16833 belongs to lineage 2, they were genetically distinct in the study. Frameshifts in the 6-guanine homopolymer from nt193 to 198 were previously reported in several M. tuberculosis clinical isolates (13, 16, 23, 24) with no apparent links to our local cases, indicating their independent acquisition of this mutation. After exposure to low concentrations of BDQ (0.03 μg/mL) or CFZ (0.12 μg/mL) for 3 or 4 weeks, both H37Rv and clinical isolate 16833 harbored *Rv0678* mutations, but no *atpE* mutations were found. One common *Rv0678* mutant with c244t was found in spontaneous mutation and after incubation with CFZ. Selection by CFZ may explain the occurrence of *Rv0678* mutations in some BDQ-resistant isolates from TB patients without the prior use of bedaquiline (12, 16). The exposure of clinical *Rv0678* mutants 12657 and 13476 to a low concentration of BDQ over 4 weeks also did not result in the further selection of an *atpE* mutation or a new *Rv0678* mutation. One limitation of our study is that these findings were based on a small number of isolates. The clinical isolates that were used in this study were pre-XDR and XDR strains. In view of the widespread usage of BDQ for almost all rifampin-resistant TB, MDR/rifampin-resistant strains are also needed to examine the emergence of BDQ resistance. However, a single resistant colony with an *atpE* mutation was selected by a low concentration of BDQ in an *Rv0678* mutant with H37Rv background, suggesting that the presence of an *Rv0678* mutation could increase the risk of a subsequent *atpE* mutation upon continued BDQ exposure. The fitness costs of drug resistance mutations strongly influence the epidemiological success of drug-resistant pathogens. For example, the secondary *rpoB* mutations at positions 435 and 1,106, which are associated with higher rifampin MICs, conferred a compensatory recovery of fitness and enhanced transmission of the evolved KZN/LAM4 strain, compared to the founder strain with an isolated L452P mutation (18). For the BDQ-resistant strains, the lower frequency of *atpE* mutations in the clinical isolates potentially indicates the higher fitness costs of such mutants (15, 25), whereas *Rv0678* mutants seem to have the same fitness, in comparison to the corresponding isolate, or even a small advantage (26). *Rv0678* mutants have been identified from TB patients without prior exposure to bedaquiline or clofazimine (12, 16). Possible explanations for this observation include preexisting genetic diversity in M. tuberculosis isolates and selection by treatments with azole antifungals or other antimicrobials that are used for TB or other infections. A large scale of clinical isolates is needed to investigate the evolutionary trajectory of the emerging BDQ resistance.

*Rv0678* mutations were associated with low-level resistance (BDQ MIC of <1 μg/mL), and *atpE* mutations conferred high-level resistance with BDQ MICs of >1μg/mL. After exposure to high concentrations of BDQ (0.3 μg/mL [5× MIC] or 30 μg/mL [50× MIC]) for 3 or 4 weeks, colonies with *atpE* mutations were selected in the H37Rv background, and even more were selected in the *Rv0678* mutant background. This indicates that *atpE* mutations may be selected directly by exposure to a high concentration of BDQ without the stepwise acquisition of mutations, beginning with *Rv0678* mutations. However, consistent with the results of previous studies (13, 27), *atpE* mutations did occur alongside *Rv0678* mutations. In our study, we exposed bacteria to a single concentration of BDQ for 3 or 4 weeks. The results suggested that *atpE* mutations were selected through exposure to high drug concentrations. This may explain the occurrence of *atpE* mutations in clinical isolates without *Rv0678* mutations.

The acquired c188t, g187c, and a83g mutations in *atpE* were also previously reported in clinical isolates (13). In our study, the expression of *mmpL5* and *mmpS5*, encoding the efflux pump components, did not decrease after the *Rv0678* mutants acquired *atpE* mutations to withstand the increased drug pressure. The presence of other strong companion drugs may prevent the acquisition of high-level resistance in the form of *atpE* mutations (28, 29). In our previous studies, diarylquinolines with superior potency, such as TBAJ-587 and TBAJ-876, in combination with supporting drugs could overcome *Rv0678*-mediated resistance (29, 30). A cross-resistance between CFZ and BDQ is conferred by *Rv0678* mutations, but CFZ at a high concentration killed all of the *Rv0678* mutants, suggesting that CFZ may be a useful companion drug with which to treat patients with *Rv0678* mutants, but this is only if adequate exposures are achieved. Further study is needed to evaluate the CFZ concentrations that better match the concentrations that can be achieved in humans.

Mutations in *Rv0678* are associated with reduced susceptibility to BDQ and CFZ, and they have been identified in strains that were selected *in vitro* (10) and *in vivo* (11, 28, 31) as well as in clinical isolates (6, 12, 16). Because *Rv0678* normally represses the expression of the adjacent operon containing *mmpL5* (*Rv0676c*) and *mmpS5* (*Rv0677c*) (32), loss-of-function mutations in *Rv0678* increase the transcription of *mmpS5* and *mmpL5* (33). To make the role of each protein in the MmpL5-MmpS5 efflux transporter clear, we constructed overexpression strains and found that *mmpS5* and *mmpL5* must be overexpressed together to confer resistance to BDQ or CFZ, which supports the conclusion that the expression of MmpS5 facilitates the assembly of monomeric MmpL5 into a functional homotrimer (34). The BDQ MICs in the strain overexpressing both *mmpS5* and *mmpL5* are similar to that of the *Rv0678* mutant (L11) in this study and to those of clinical isolates with *Rv0678* mutations (35).

To further investigate the correlation between *Rv0678* and *atpE* mutation, four BDQ-resistant strains, including an *Rv0678* mutant, an *atpE* mutant, and dual mutants with *Rv0678* and *atpE* mutations were subjected to whole-genome sequencing. No other mutations were identified as being associated with BDQ resistance. A quantitative proteome analysis showed that mutants with *Rv0678* mutations upregulated MmpL5 and MmpS5 expression, which is consistent with the results of a previous study (6). We also found that secreted immunogenic proteins (MPT64, MPT70, espA, Apa, CFP-2, and Ag85B) and proteins that were involved in the biosynthesis and transport of phthiocerol dimycocerosates (PpsB, PpsC, PpsD, and DrrB) were upregulated in the *Rv0678* mutants. In line with the results of previous studies, MmpL and MmpS proteins mediate the transport of important cell wall lipids, including phthiocerol dimycocerosate (36). Interestingly, the BDQ-resistant strains, including the *atpE* mutant, also upregulated the iron homeostasis protein HupB, which regulates *Rv0678*, *mmpS5* (37), and *SodB* (38) in M. tuberculosis. The MmpL5-MmpS5 efflux pump has been linked to the export of nonferrated siderophores to the extracellular environment (39). Considering its roles in drug efflux and siderophore export, these results suggest that MmpL5-MmpS5 can be regarded as a potential target for antituberculosis drug development.

The upregulated nucleoid-associated protein mIHF as well as ribosomal proteins need to be further validated with regard to their association with BDQ resistance, but they were previously shown to be differentially abundant upon exposure to BDQ (40). A study in preprint suggested that CFZ inhibits the binding of mIHF to DNA and interferes with mycobacterial gene expression (41). BDQ-treated M. tuberculosis redirects the central carbon metabolism to become dependent on glycolysis for ATP production, operates a bifurcated tricarboxylic acid cycle by increasing the flux through the glyoxylate shunt, and requires enzymes of the anaplerotic node and methylcitrate cycle (42). In our study, some major upregulated proteins (GpsA, GlgM, and Ald) may correlate with the M. tuberculosis cell metabolism in the BDQ-resistant strains with *atpE* mutations only, but not *Rv0678* mutations. Glycerol-3-phosphate dehydrogenase (GpsA) is a glycolytic enzyme. α-maltose-1-phosphate synthase GlgM is involved in the biosynthesis of the maltose-1-phosphate building block that is required for alpha-glucan production by the key enzyme GlgE (43). Alanine dehydrogenase (Ald) converts pyruvate to alanine as well as glyoxylate to glycine, and this is concurrent with the oxidation of NADH to NAD. It is involved in the metabolic remodeling of M. tuberculosis through its possible interactions with both the glyoxylate and methylcitrate cycles (44). Further studies are warranted to investigate how mutations in BDQ-resistant strains impact the cellular metabolism.

Our work highlights the urgent need for extensive surveillance for *Rv0678* and *atpE* mutations in association with the clinical usage of BDQ to reduce the risk of treatment failure and limit the epidemiological success of BDQ-resistant strains.

## MATERIALS AND METHODS

### Clinical isolates.

Three clinical isolates (strains 16833, 12657, and 13476) were collected from individual TB patients who attended Beijing Chest Hospital. All three clinical isolates, including the *Rv0678* mutants, belonged to the Beijing genotype, based on the results of whole-genome sequencing and analysis, as previously described (12). The XDR-TB isolate 16833 was resistant to INH, RIF, STR, EMB, OFX, LVX, CAP, AMK, ETO, and PAS, but it was susceptible to BDQ (12). Both the pre-XDR isolate 12657 and the XDR isolate 13476 were bedaquiline-resistant strains harboring *Rv0678* mutations (12).

### Construction of gene overexpression strains.

The mmpS5 (429 bp), mmpL5 (2,895 bp), and mmpS5-mmpL5 (3,320 bp) genes were PCR-amplified from genomic H37Rv DNA (12) by using the primers described in Table S1 and cloned into the pMV361 vector at the EcoRI and HindIII sites. The resulting recombinant plasmids, namely, pMV361-L5, pMV361-S5, pMV361-L5-S5, were checked via sequencing and were introduced, along with the empty pMV361 vector, into wild-type H37Rv via electroporation. Recombinant strains were selected on Middlebrook 7H10 containing 50 μg/mL kanamycin after 4 weeks of incubation at 37°C and were subsequently grown in liquid medium.

### Real-time PCR.

RNA was isolated from MTB strains by using a RiboPure-Bacteria Kit (Ambion, AM1925). The quantity and quality of each RNA sample was determined using a Denovix spectrophotometer. Reverse transcription was performed with a PrimeScript RT Reagent Kit with gDNA Eraser (TaKaRa). Quantitative PCR was performed with a StepOnePlus real-time PCR system (Applied Biosystems), using SYBR Premix *Ex Taq* II (TaKaRa, Japan) with gene-specific primers (Table S1), as previously described (45). The relative quantification was calculated by using the 2^−ΔΔCt^ method with *sigA* as the internal control (45).

### MIC determination.

The MICs of BDQ and CFZ were determined via microplate alamarBlue assays (MABA), using twofold dilutions, as described previously (12, 31). The solvent for BDQ and CFZ was dimethyl sulfoxide (DMSO). The MIC was defined as the lowest concentration that resulted in a reduction in fluorescence of ≥90%, relative to the mean fluorescence of the replicate drug-free controls. The experiments were performed in duplicate, and the mean values are shown.

### Measurement of the frequency of spontaneous resistance.

M. tuberculosis strains were cultured in Middlebrook 7H9 broth (Difco, USA) supplemented with 0.2% (vol/vol) glycerol, 0.05% Tween 80, and 10% (vol/vol) oleic acid-albumin-dextrose-catalase (OADC) (Becton, Dickinson, USA). A total of 10^8^ CFU of log-phase bacteria were plated onto selective agar containing 0.125, 0.25, 0.5, or 1 μg/mL of BDQ. The number of total CFU was estimated by making serial dilutions and plating onto nonselective Middlebrook 7H10 agar. The concentrations of the solvent DMSO that were used in the controls (0.1% vol/vol) were the same as those that were used in the treatment group. After 4 weeks of incubation, the colonies were counted, and the frequency of spontaneous resistance was calculated.

### Mutant selection under different concentrations of BDQ or CFZ.

The MICs of BDQ and CFZ against wild type M. tuberculosis are approximately 0.06 μg/mL and 0.24 μg/mL, respectively. Cultures containing approximately 10^7^ CFU/mL were exposed to BDQ at 0.03 μg/mL (1/2× MIC), 0.3 μg/mL (5× MIC), and 30 μg/mL (500× MIC) or to CFZ at 0.12 μg/mL (1/2× MIC) and 120 μg/mL (500× MIC) in 7H9 medium for 3 or 4 weeks. After incubation, aliquots of each culture were plated on drug-free agar and agar containing BDQ at 0.125 μg/mL and 1 μg/mL. The latter concentration is just above the mean (±SD) steady-state plasma concentration of BDQ, that is, 0.902 ± 0.535 μg/mL, in TB patients at week 8 of treatment, using the approved dosing regimen (46). The average steady-state CFZ plasma concentration was estimated to be 0.36 μg/mL with a dosage of 100 mg/day (47).

### Whole-genome sequencing and DNA sequencing.

Genomic DNA from M. tuberculosis was isolated and subjected to PCR amplification using the *Rv0678*, *pepQ*, and *atpE* primers, and the PCR products were sequenced (12). The *Rv0678* and *atpE* mutants were sequenced using an Illumina HiSeq 4000 (Illumina, Inc.) for the whole-genome sequencing, with an expected depth of 40× and a read length of 300 bp. The genome assembly was performed using the SOAPdenovo V2.04 software package, and the reads were aligned with the reference sequence of MTB H37Rv (NC_00962.3) by using SOAPaligner v2.21, as described previously (12). The whole-genome alignments for the single nucleotide polymorphism (SNP) analysis were generated using MUMMER v 3.22. The sequences with lengths of 100 bp on both sides of the SNP in the reference sequence were extracted and aligned with the assembly results to verify the SNP sites by using BLAT v34. The BLAST, TRF, and RepeatMasker software tools were used to predict the SNPs in repeat regions. For the insertion-deletion (InDel) analysis, the samples and the reference sequence were aligned by using the LASTZ software package. The preliminary InDel results were obtained via processing with the axt_correction, axtSort, and axtBest programs. The InDel sites were aligned with sequencing reads of 150 bp in the upstream and downstream of the reference sequence. The alignment results were verified using BWA and SAMtools.

### Proteome analysis.

The protein extraction and analysis were performed as previously described (48). Briefly, mycobacterial cell extract was prepared in lysis buffer (7 M urea, 2 M thiourea, 0.1% CHAPS) and was ground with glass beads for 20 min. Cell debris were removed via centrifugation, and the supernatant was collected and measured via a Bradford protein assay. After reduction and digestion, the peptides were labeled via the TMT labeling method. The samples were mixed and lyophilized. The acquired peptide fractions were loaded on a nano UPLC-MS/MS system that consisted of a RIGOL L-3000 HPLC system and an Orbitrap Fusion Lumos mass spectrometer (Thermo Scientific). The raw MS/MS data were analyzed using Proteome Discoverer v2.4 by Beijing Qinglian Biotech, Co., Ltd. The database that was used in this experiment is the UniprotKB 2021_02 M. tuberculosis H37Rv database.

### Statistical analysis.

The differential significance test in the proteome analysis was performed via the MaxQuant significance A approach. The differential proteins of the two groups were selected for further analysis with a *P* value of ≤0.05 and a difference ratio of ≥1.2.
