# Supplementary material for: Bedquiline Resistance Mutations: Correlations with Drug Exposures and Impact on the Proteome in M. tuberculosis
Source: Antimicrob Agents Chemother. 2023 May 31;67(7):e01532-22. doi: 10.1128/aac.01532-22 (PMC10353445; doi:10.1128/aac.01532-22)
Supplement: Supplemental file 1 — Supplemental material. Download aac.01532-22-s0001.pdf, PDF file, 0.25 MB [file aac.01532-22-s0001.pdf]

Supplementary materials

Table S1 Oligonucleotides used in this study.

Primer	Sequence (5’-3’)
Mmpl5-F	CCGGAATTCATGATCGTGCAAAGGAC (EcoRI)
Mmpl5-R	CCTCAAGCTTTCAGACCAAGGCGAAG (HindIII)
Mmps5-F	CCGGAATTCATGATTGGGAACTCTCAAG (EcoRI)
Mmps5-R	CCTCAAGCTTTCATGCGGATTTCACAAAG (HindIII)
Rv0676c-(RT)F	CCAACGGCACACCGAGGGTC
Rv0676c-(RT)R	CAGGACCGGGAGTCGGCGTA
Rv0677c-(RT)F	GCCATCGACGGTGATTCGGCA
Rv0677c-(RT)R	GACCTCGACGCCAAGCCACAG
Rv0678c-(RT)F	TCCGGGCAATGGCCGAACTG
Rv0678c-(RT)R	GTCCTCTCCGGTTCGCTGGC
SigA-(RT)F	CGGCTACGCTTCGGCCTTAC
SigA- (RT)R	GGCGCAACTTCGACATAGTCTTG
Rv0678-F	TGCCTTCGGAACCAAAGAA
Rv0678-R	GACAACACGGTCACCTACAA
atpE-F	TGTACTTCAGCCAAGCGATGG
atpE-R	CCGTTGGGAATGAGGAAGTTG
pepQ-F	ATCAATGCCCCCTGGAAC
pepQ-R	GCACGTTCTTCAACTTGGTG

F and R stand for forward and reverse, respectively.

Table S2 Ten proteins with the lowest average fold change across all 4 mutants L11, H3, D1 and D12 compared to H37Rv.

Rv number	Gene Name	Description	FC(L11/Rv)	FC(H3/Rv)	FC(D1/Rv)	FC(D12/Rv)
Rv3402c	*Rv3402c*	Protein Rv3402c	0.36	0.45	0.37	0.4
Rv0491	*regX3*	Sensory transduction protein RegX3	0.38	0.38	0.36	0.36
Rv2623	*Rv2623*	Universal stress protein Rv2623	0.4	0.49	0.44	0.48
Rv2428	*ahpC*	Alkyl hydroperoxide reductase C	0.41	0.46	0.4	0.44
Rv2031c	*hspX*	Alpha-crystallin	0.44	0.48	0.42	0.48
Rv0080	*Rv0080*	Uncharacterized protein Rv0080	0.46	0.61	0.54	0.56
Rv2133c	*Rv2133c*	Uncharacterized protein	0.48	0.57	0.57	0.58
Rv2032	*acg*	Putative NAD(P)H nitroreductase acg	0.48	0.61	0.55	0.58
Rv3134c	*Rv3134c*	Universal stress protein Rv3134c	0.49	0.59	0.52	0.58
Rv0097	*Rv0097*	Putative dioxygenase Rv0097	0.5	0.62	0.39	0.48

Table S3 Ten proteins with the lowest average fold change across the 3 *Rv0678* mutants L11, D1 and D12 but not H3 compared to H37Rv.

Rv number	Gene Name	Description	FC(L11/Rv)	FC(H3/Rv)	FC(D1/Rv)	FC(D12/Rv)
Rv1813c	*Rv1813c*	Uncharacterized protein Rv1813c	0.49	0.89	0.50	0.54
Rv0678	*mmpR5*	HTH-type transcriptional regulator MmpR5	0.52	1.33	0.52	0.63
Rv3140	*fadE23*	Probable acyl-CoA dehydrogenase FadE23	0.52	0.88	0.63	0.60
Rv0196	*Rv0196*	Uncharacterized HTH-type transcriptional regulator Rv0196	0.58	0.86	0.73	0.78
Rv0064	*Rv0064*	UPF0182 protein Rv0064	0.59	0.84	0.61	0.54
Rv2378c	*mbtG*	L-lysine N6-monooxygenase MbtG	0.61	0.81	0.66	0.70
Rv1142c	*echA10*	Probable enoyl-CoA hydratase EchA10 (Enoyl hydrase) (Unsaturated acyl-CoA hydratase) (Crotonase)	0.62	0.80	0.64	0.71
Rv2738c	*Rv2738c*	Signal transduction histidine kinase	0.62	0.82	0.69	0.75
Rv0197	*Rv0197*	Possible oxidoreductase	0.64	0.87	0.62	0.69
Rv1239c	*corA*	Magnesium transport protein CorA	0.64	0.84	0.69	0.72

Table S4 Ten proteins with the lowest average fold change in the *atpE* mutant only (H3) but not *Rv0678* mutant L11 compared to H37Rv.

Rv number	Gene	Description	Fold Change H3/Rv	Fold Change L11/Rv	Fold Change D1/Rv	Fold Change D12/Rv
Rv0572A	*Rv0572A*	Uncharacterized protein Rv0572A	0.6	0.7	0.8	0.7
Rv2949c	*Rv2949c*	Chorismate pyruvate-lyase	0.6	0.8	0.7	0.7
Rv2231c	*Rv2231c*	Uncharacterized aminotransferase Rv2231c	0.6	0.7	0.7	0.8
Rv2002	*fabG3*	3-alpha-(or 20-beta)-hydroxysteroid dehydrogenase	0.6	0.7	0.7	0.7
	Rv2049c	Uncharacterized protein	0.7	0.8	0.7	0.8
Rv1630	*rpsA*	30S ribosomal protein S1	0.7	0.7	0.6	0.7
Rv3285	*accA3*	Probable bifunctional protein acetyl-/propionyl-coenzyme A carboxylase (Alpha chain) AccA3	0.7	0.8	0.7	0.7
Rv0005	*gyrB*	DNA gyrase subunit B	0.7	0.8	0.7	0.7
Rv2533c	*nusB*	Transcription antitermination protein NusB	0.7	0.7	0.7	0.7
Rv2038c	*Rv2038c*	Probable sugar-transport ATP-binding protein ABC transporter	0.7	1.2	0.9	0.7
